# Fear extinction retention in children, adolescents, and adults

**DOI:** 10.1016/j.dcn.2025.101509

**Published:** 2025-01-09

**Authors:** Ebba Widegren, Johan Vegelius, Matilda A. Frick, Ashika A. Roy, Stefan Möller, Johan Lundin Kleberg, Johanna Motilla Hoppe, Olof Hjorth, David Fällmar, Daniel S. Pine, Karin Brocki, Malin Gingnell, Andreas Frick

**Affiliations:** a Department of Medical Sciences, Experimental Cognitive and Affective Neuroscience Lab, Uppsala University, Uppsala, Sweden; b Department of Medical Sciences, Child and Adolescent Psychiatry, Uppsala University, Uppsala, Sweden; c Department of Psychology, Stockholm University, Stockholm, Sweden; d Department of Psychology, Lund University, Lund, Sweden; e Department of Psychology, Uppsala University, Uppsala, Sweden; f Department of Surgical Sciences, Neuroradiology, Uppsala University Hospital, Uppsala, Sweden; g Section on Development and Affective Neuroscience, National Institute of Mental Health Intramural Research Program, Bethesda, MD, USA

**Keywords:** Fear conditioning, Threat conditioning, Development, Fear extinction, Fear retention, FMRI

## Abstract

Past results suggest that fear extinction and the return of extinguished fear are compromised in adolescents. However, findings have been inconclusive as there is a lack of fear extinction and extinction retention studies including children, adolescents and adults. In the present study, 36 children (6–9 years), 40 adolescents (13–17 years) and 44 adults (30–40 years), underwent a two-day fear conditioning task. Habituation, acquisition, and extinction were performed on the first day and an extinction retention test > 24 h later. Skin conductance responses were recorded during all phases of fear conditioning and functional magnetic resonance imaging (fMRI) was conducted during the fear retention test. All groups acquired and extinguished fear as measured with SCR, with no group differences in SCR during extinction retention. The groups had largely similar neural fear responses during the retention test, apart from adolescents displaying stronger amygdala fear response than children, with no differences between adolescents and adults. The findings do not support an adolescent extinction dip, and there was only marginal evidence of progressive changes in fear conditioning across development. In contrast to findings in rodents, fear conditioning in humans may elicit similar physiological responses and recruit similar neural networks from childhood to adulthood.

## 1 Introduction

Fear enables appropriate responding to danger. Consistent with this view, learning the identity of cues that signal danger manifests similarly across species and supports survival. Likewise, when threat cues no longer reliably predict danger, an individual needs to update their learned associations. In a laboratory setting, Pavlovian fear conditioning models this form of learning (Lonsdorf et al., 2017). During acquisition, fear is learned when a neutral stimulus predicts the occurrence of an inherently aversive stimulus (unconditioned stimulus; US), leading the previously neutral stimulus to become a conditioned stimulus (CS+) that elicits conditioned fear responses. This can manifest in many signals, including physiological, such as skin conductance responses (SCR), affective ratings of CS and US expectancy. It can also be detected in brain activity, as measured by functional magnetic resonance imaging (fMRI) (Björkstrand et al., 2022, Frick et al., 2022, Ojala and Bach, 2020). Most human studies use differential cue fear conditioning, adding a conditioned stimulus (CS-) never paired with the US, in addition to the CS+ . Following acquisition, many paradigms include an extinction learning phase and sometimes also an extinction retention test (Lonsdorf et al., 2019a) During extinction learning, the CS appears without the US, resulting in fear attenuation. Following extinction learning, the retention test can be performed to investigate remaining fear response to the CS (Bach et al., 2023).

The current view is that extinction learning does not replace the original fear memory, but rather results in the creation of a new ‘safety memory’ that inhibits the fear response (Craske et al., 2014). The response to the CS during the retention test is thus thought to reflect the relative dominance of the ‘safety’ versus the ‘fear’ memory after the passage of time and/or change in context (Lonsdorf et al., 2019b), and as such is sometimes referred to as return of fear. The retention test phase may be the most clinically relevant, as it relates closely to the goal of exposure-based treatments to decrease fear responding not just within session, but over time and across contexts (Craske et al., 2018, Vervliet et al., 2013). Moreover, since extinction learning measures may not predict fear responding at a retention test (Prenoveau et al., 2013), they may provide limited insights on persistent or recurring fear.

The neural correlates underlying fear conditioning have been excellently reviewed by others (Fullana et al., 2016, Fullana et al., 2018). The main brain regions involved in acquisition, extinction learning and extinction retention include the amygdala, ventromedial prefrontal cortex (vmPFC), dorsal anterior cingulate cortex (dACC), hippocampus, and insula. The amygdala is thought to be pivotal in fear conditioning, as demonstrated by animal studies (LeDoux, 2000) and shown in human neuroimaging studies (Wen et al., 2022), although meta-analyses have failed to find consistent fear-related amygdala activity using fMRI (Fullana et al., 2016, Fullana et al., 2018). Fear extinction learning and extinction retention have been specifically linked to the vmPFC and its connectivity to the amygdala, as well as to the hippocampus (Milad et al., 2007, Milad and Quirk, 2012, Morriss et al., 2019, Phelps et al., 2004). However, both vmPFC and amygdala involvement in fear extinction has been debated, with a recent meta-analysis of studies on the neural correlates of extinction learning and extinction retention failing to show consistent activation in either of these regions (Fullana et al., 2018). The authors suggest that this may be due to study protocols with weak USs and the lack of studies using an unextinguished CS. Instead, the meta-analysis showed consistent activation in the insula and the dorsal anterior cingulate cortex during extinction learning and extinction retention, regions thought to be involved in threat appraisal and the expression of fear states.

Despite considerable interest (Grasser and Jovanovic, 2021, Klein et al., 2021, Pattwell et al., 2012, Shechner et al., 2014), little is known regarding how development affects fear learning in humans (Stenson et al., 2023). Human infants effectively learn to fear conditioned simple stimuli (Ingram and Fitzgerald, 1974), but discriminatory conditioning develops in pre-school or early school age (Gao et al., 2010). Some previous studies reveal an adolescent dip in extinction learning and extinction retention, first demonstrated in rats (Kim and Richardson, 2008) and later partially translated to humans by Pattwell et al. (2012). Yet, more recent findings are mixed, with some studies suggesting a progressive change in fear conditioning processes, some reporting no age differences, and others reporting a dip specifically in adolescence (Britton et al., 2013, Den et al., 2015, Ganella et al., 2018, Gao et al., 2010, Morriss et al., 2019, Shechner et al., 2015a, Shechner et al., 2015b, Waters et al., 2017). It should also be noted that the largest study to date investigating age-related differences in fear acquisition and extinction, involving 351 participants aged 8–50 years, found no associations between age and fear extinction learning using physiological outcomes (Abend et al., 2020). However, similar to Pattwell et al. (2012), this study did not include an extinction retention test. Another study reported impaired extinction learning and extinction retention in adolescents compared to children and adults using affective ratings, but they did not include more objective physiological measures (Waters et al., 2017). Remarkably, there are no studies examining the proposed adolescent deficit in extinction retention including participants from childhood to adulthood using physiological or neural responses.

Adolescence is characterized by changes in connectivity between the amygdala and cortical regions, such as the vmPFC, which may contribute to age-related differences in fear conditioning (Casey et al., 2019, Kitt et al., 2023, Morriss et al., 2019). Frontal cortical areas, such as the vmPFC, may regulate subcortical regions such as the amygdala, in ways that do not mature until late in adolescence (Gabard-Durnam et al., 2014). In support of this view, amygdala-PFC connectivity changes across development, with a clear transition around 10 years of age (Gabard-Durnam et al., 2014, Gee et al., 2013). Indeed, previous studies have shown that compared to adults, adolescents have increased fear-related (CS+>CS-) amygdala activity and delayed recruitment of the mPFC during extinction learning (Morriss et al., 2019) and reduced vmPFC activity during extinction retention (Ganella et al., 2018).

Hence, mixed evidence reveals a developmental dip in fear extinction learning potentially related to development of the PFC. However, studies investigating neural correlates of fear extinction learning and fear extinction retention in different age groups are rare, and to our knowledge, there have been no studies investigating extinction retention using physiological or neural measures across development from childhood to adulthood. It remains unknown whether the results from rodent studies can be extended to a human population, and if the neural alterations described in extinction learning and retention studies are specific to the adolescent period or whether they hold true for children as well. Additionally, adolescence is known to be a developmental phase characterized by an increased vulnerability to develop anxiety and depression, disorders thought to be related to fear conditioning mechanisms (Britton et al., 2011). Thus, studies investigating the neural correlates of fear conditioning across development are needed, both to elucidate how brain development affects fear conditioning processes but also to understand some of the neural mechanisms underlying adolescent vulnerability to develop depression and anxiety. In this vein, the present work aimed to test developmental changes in fear conditioning. We employed SCR, affective ratings, and fMRI to test the hypothesis of an adolescent dip in extinction learning and extinction retention. In addition, we also tested for progressive changes in fear conditioning processes from childhood to adulthood.

## 2 Method

### 2.1 Recruitment

Children (6–9 years), adolescents (13–17 years) and adults (30–40 years) were recruited from existing longitudinal studies at the Department of Psychology, Uppsala University, and through public advertisements. Adult participants and caregivers of children and adolescents provided written informed consent, and children and adolescents provided informed assent to participate in the study. Participants were screened for eligibility through online questionnaires, filled out by either the participants themselves or, in the child and adolescent groups, by their caregivers. Exclusion criteria included atypical development, contraindications for magnetic resonance imaging, uncorrected vision or hearing impairment, pregnancy, illicit drug use or use of psychotropic medication. Atypical development was defined as premature birth (before 37 weeks), developmental abnormalities, or presence of any medical, neurological or psychiatric condition such as ADHD, autism, neurological disorder. After initial screening, participants also underwent assessment by a trained clinician using the Mini International Neuropsychiatric Interview (MINI; Sheehan et al., 2010) and participants with current psychiatric illness or history of severe psychiatric illness were excluded. The study was approved by the Swedish Ethical Review Authority (2019–01929, 2022–02234–02). Participants were reimbursed with a gift card worth about $85 for their participation.

### 2.2 Procedure

Included participants came for two visits. Prior to the first visit, adults and adolescents and the caregivers of children and adolescents filled in additional online questionnaires, including educational level (0: *elementary school* to 4: *college/university degree*) and yearly income (0: *0–$10,000 to 5: >$50,000)*. For adults, participant SES was calculated as the sum of the educational level and yearly income. For children and adolescents, SES was first calculated for both parents, and the average of these was used as an indicator of household SES. The first visit took place in the lab, where participants underwent fear acquisition and extinction learning. The second visit (>24 h and <2 weeks later) was a scanning session at the MR scanner where participants underwent the fear retention test. Both visits included additional data collection and blood sampling for analyses of hormone levels, not reported here.

### 2.3 Fear conditioning protocol

**Fig. 1:**
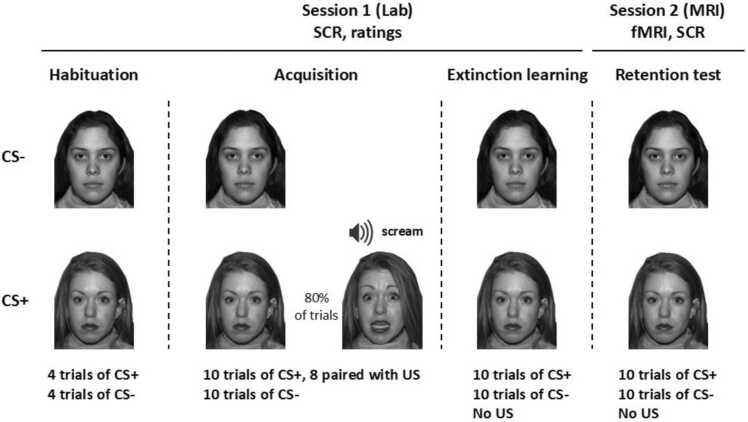
Illustration of the fear conditioning task. In session 1, participants underwent habituation, fear acquisition and extinction learning in the lab, and in session 2, > 24 h later, the extinction retention test was performed inside the magnetic resonance imaging (MRI) scanner. The conditioned stimulus (CS) that was paired with the unconditioned stimulus (US) scream (CS+) was counterbalanced across participants.

The fear conditioning protocol used was an adapted version of the Screaming Lady paradigm (Lau et al., 2008), a differential cued conditioning paradigm previously used in child and adolescent populations (Britton et al., 2013, Chauret et al., 2019, Gold et al., 2020). The paradigm consisted of four phases of conditioning: habituation, acquisition, extinction learning and a retention test. The stimuli used included two photographs of actresses with neutral facial expressions used as conditioned stimuli (CS+ and CS-). The aversive unconditioned stimulus (US) was a photograph of the same actress as the CS+ displaying a fearful facial expression, instead of a neutral, paired with the sound of a scream (see Fig. 1). The stimuli constituting the CS+ and CS- were balanced across participants.

The habituation phase consisted of 4 presentations each of CS+ and CS- for 7 s. Directly following the habituation, fear acquisition was conducted using 10 CS+ and 10 CS- presentations, with 8 of the 10 CS+ directly followed by the US (80 % reinforcement rate). The CS+ was presented for 6 s, the US for 1 s, and the CS- for 7 s. During extinction learning and the retention test, 10 CS+ and 10 CS- were presented for 7 s each, without any US presentations. All CS+ and CS- presentations were done in pseudorandom order with no more than two of each type presented in a row. Inter-trial interval for all phases consisted of a black fixation cross on a white background displayed for a variable time sampled from a Gaussian distribution with a mean of 12 s and SD 1 s.

The sound volume used for the US was individually calibrated in a step-wise fashion using the sound of a ringing bell to reach a volume that was ‘unpleasant, but tolerable’ for adolescents and adults, but set to a fixed volume for 32 of the children. Initially, we used an individual setting for the children, but abandoned this after several children discontinued the task. Hence, 4 children made individual calibrations, and 32 children had fixed volumes. The volume that was pre-set for children was also considered ‘unpleasant, but tolerable’, and the specific volume was decided during piloting of the paradigm, where children who chose a higher volume during the calibration found the paradigm too aversive.

Habituation and acquisition were performed with no pause in between. After acquisition, participants performed approximately 90 min of cognitive testing, including a 15-min break, and then they completed the extinction learning phase. The retention test was performed in the MRI scanner during visit 2.

### 2.4 Measures

#### 2.4.1 Skin conductance

Skin conductance was recorded using BIOPAC MP160 (during fear habituation, acquisition, and extinction learning phases in the lab) and BIOPAC MP150 (during the retention test in the MR scanner). Disposable electrodes were placed on the hypothenar eminence on the left hand of the participants. The electrodes were prepared with isotonic gel prior to application. A hardware 10 Hz lowpass filter was applied to the skin conductance signal. The skin conductance data was first visually inspected to control for recording errors and then analyzed using in-house MATLAB scripts with the following steps: median filtering using a 10 ms window length, bandpass filtering using a first order Butterworth filter (0.03–5 Hz), down sampling to 100 Hz, and automatic extraction of baseline-corrected peak skin conductance responses (SCR) 1–5 s post stimulus onset. The baseline level was set to the mean skin conductance level 0–1 s post stimulus onset. SCR < 0.01 microsiemens was set to 0 and SCR > 5 was set as missing values as these were deemed as physiologically implausible. All SCR scores were subsequently square-root transformed.

#### 2.4.2 Affective ratings

Subjective levels of fear were assessed using a visual analog scale (VAS), displaying faces ranging from happy to fearful, with numbers displayed below each face, ranging from 1 (not fearful at all) to 5 (very afraid). Participants in the study were asked to rate how fearful they felt when viewing the CS+ and CS- before the habituation phase, after acquisition, and after extinction learning.

#### 2.4.3 Contingency awareness

After the extinction learning phase, participants were asked to indicate if the CS+ face and the CS- face screamed during the session. Participants who reported that the CS+ face, but not the CS- face, screamed were deemed to be contingency (CS-US) aware.

#### 2.4.4 Magnetic resonance imaging

During session 2, magnetic resonance imaging (MRI) was performed using a Philips Achieva 3.0 T whole body MR-scanner (Philips Medical Systems, Best, The Netherlands) equipped with a 32-channel head-coil. An anatomical T1-weighted image (echo time (TE)=3.8ms; repetition time (TR)=8.2ms; inversion time=685.5ms; flip angle=8°; field of view=240 × 240 mm^2^; voxel size=1 × 1 × 1 mm^3^; 220 contiguous slices) was used for anatomical referencing. During the extinction retention test, a blood-oxygenation-level dependent (BOLD) echo planar imaging (EPI) sequence was acquired (TE=30ms; TR=2000ms; flip angle=90, acquisition matrix=64 ×64, voxel size=3.0 ×3.0 ×3.0 mm^3^, gap=0.9 mm, 32 interleaved axial slices). Visual stimuli were presented through goggles mounted on the head coil (Visual System,NordicNeuroLab, Bergen, Norway) using the Psychtoolbox in Matlab (Natick, Massachusetts, USA). Morphological images from all subjects were reviewed by a senior consultant in neuroradiology (DF) to exclude malformations and significant parenchymal defects.

### 2.5 Functional magnetic resonance imaging analysis

Preprocessing was performed using *fMRIPrep* 23.1.4 (Esteban et al., 2019; RRID:SCR_016216), which is based on *Nipype* 1.8.6 (Gorgolewski et al., 2011; RRID:SCR_002502). See Supplemental Material for details. Briefly, the following preprocessing was performed. First, a reference volume and its skull-stripped version were generated using a custom methodology of fMRIPrep. The BOLD reference was then co-registered to the T1w reference using bbregister (FreeSurfer) which implements boundary-based registration. Co-registration was configured with six degrees of freedom. Head-motion parameters with respect to the BOLD reference (transformation matrices, and six corresponding rotation and translation parameters) are estimated before any spatiotemporal filtering using mcflirt (FSL 5.0.9). BOLD runs were slice-time corrected using 3dTshift from AFNI 20160207 (RRID:SCR_005927). The BOLD time-series were resampled onto their original, native space by applying the transforms to correct for head-motion and resampled into standard space, generating a preprocessed BOLD run in MNI152NLin2009cAsym space with isotropic 2 mm voxels. BOLD data was then spatially smoothed with an isotropic, Gaussian kernel of 8 mm FWHM (full-width half-maximum) using fslmaths (FSL) with a sigma of 3.4. Framewise displacement (FD) was computed following Power et al. (2014) using the absolute sum of relative motions. Volumes with FD > 0.9 mm were deemed to be outliers and subsequently censored in the first-level model.

Neural activity during fear retention was modeled in Statistical Parametric Mapping (2006) (SPM12; www.fil.ion.ucl.ac.uk/spm). The first-level model for each participant was fitted with onsets and durations of CS+ and CS-, convolved with the canonical haemodynamic response function from SPM12, together with the following regressors: 6 realignment parameters from the realignment step, 6 aCompCor components, and one regressor per censored volume based on FD > 0.9 mm censoring. Two sets of contrast images were created for subsequent analyses of the fear memory retention, CS+ minus CS- for all trials and for the initial two trials. Second-level activity analyses were performed with SPM12 and included whole-brain and region of interest (ROI) analyses. ROIs were based on the previous literature, defined according to the Harvard-Oxford Structural Atlas (RRID:SCR_001476), and included the amygdala, insula, hippocampus, ACC and vmPFC (medial frontal cortex). We performed analyses within the whole sample and pairwise comparisons of age groups. For these analyses we used family-wise error corrected (FWE) *p*_FWE_< .05 as our statistical threshold of significance.

Functional connectivity during fear retention was analyzed as generalized psychophysiological interactions (gPPI) using Conn version 22a (RRID: SCR_009550) (Nieto-Castanon and Whitfield-Gabrieli, 2022). Preprocessed T1-weighted structural images and smoothed BOLD functional images were directly imported to Conn together with onset files from the Screaming Lady task to contrast connectivity during CS+ presentations from connectivity during CS- presentations. Regressors for white matter, CSF, realignment parameters, as well as effects of CS+ and CS- were added to the gPPI model together with censoring regressors (>0.9 mm FD). A bandpass filter of 0.008 to infinity was selected and linear detrending was carried out. We then performed seed-to-voxel functional connectivity analyses using gPPI with the whole brain as target and with the ROIs defined above as seeds: amygdala, insula, hippocampus, dACC and vmPFC. As for the activity analyses, we performed whole-sample analyses as well as pairwise comparisons of age groups. Results were thresholded using cluster-level false discovery rate (FDR) *p*_FDR_< .05 together with a cluster-forming threshold of uncorrected *p* < .001.

### 2.6 Statistical analysis

All statistical analyses of demographics, SCR and affective ratings were performed using R 4.4.1 (R Core Team, 2024). Fear conditioning SCR was analyzed phase-wise using linear mixed-effects models from the package LmerTest (Kuznetsova et al., 2017), with SCR as outcome and including subject as a random intercept, and CS (CS+ or CS-) and trial and their interaction as fixed effects in the whole sample analyses. For group comparisons, age group was added to the model as a fixed effect together with its interaction with the other fixed effects, using adolescents as the reference group. Additionally, LMM analyses using children as the reference group were completed to compare the child group to the adult group, in order to test for progressive changes in fear conditioning processes across development. Fear extinction retention was also analyzed using the CS+ minus CS- difference in SCR for the mean response during the first two trials. For this, we fitted a linear regression model including SCR as outcome and group as predictor. Habituation in SCR to the US during the acquisition phase was investigated with linear mixed-effects model including subject as random intercept, and trial and group and their interaction as regressors. Group and phase differences in affective ratings were analyzed using cumulative link mixed models (CLMM) from the package ordinal (github.com/runehaubo/ordinal), as the data was nonparametric, with subject as random intercept and CS, phase and group and their interactions as fixed effects. Sex distributions in the groups were compared using Chi2-tests.

### 2.7 Sensitivity analyses

We performed a series of sensitivity analyses for the SCR and brain activity results by adding covariates to the models to account for SES, US sound level, and the number of days between session 1 and 2. Finally, we performed sensitivity analyses including only the participants who were CS-US contingency aware.

## 3 Results

### 3.1 Participants

**Fig. 2:**
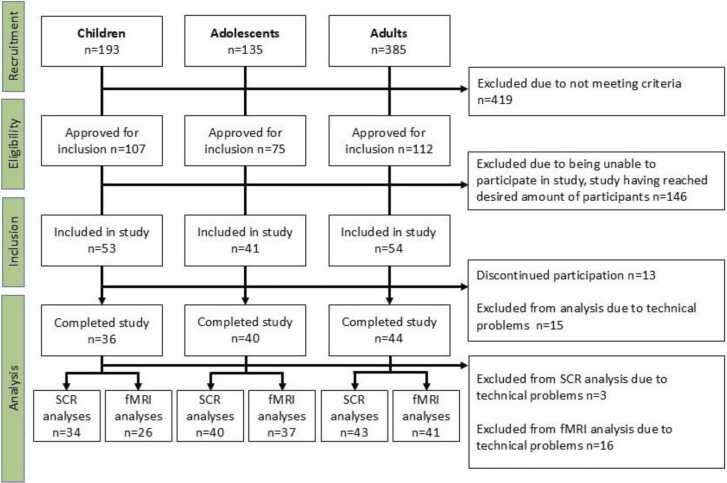
Flow chart of inclusion/exclusion of participants.

A total of 120 participants completed the study. 36 children, 40 adolescents, and 44 adults (Fig. 2 and Table 1). The groups had similar sex distributions, but differed on SES. Parents of adolescents had higher SES than adults and parents of children, with the latter two being similar. Time between the two sessions and time of day for the retention test were similar between groups. One adult had 31 days between the two sessions, all other participants underwent the second session 1–13 days after the first session. US sound level (% of max) differed between groups, children had the lowest, adolescents in the middle, and adults the highest. The groups did not differ in the proportion of participants who were CS-US contingency aware.

**Table 1:** Participant characteristics and descriptive statistics.

	Children (n = 36)	Adolescents (n = 40)	Adults (n = 44)	statistic	*p*
Age, years	8.0 (0.8)	13.7 (1.2)	34.7 (3.3)		
Range	6–9	13–17	30–40		
Sex n (%) female	22 (61 %)	21 (53 %)	23 (52 %)	χ^2^(2) = 0.78	.678
Socioeconomic status^a^	7.0 (1.7)	7.7 (1.1)	7.1 (1.6)	*F*(2114)= 2.19	.082
Range	2.5–9	5–9	2–9		
Children vs adolescents				*t(59.6)*= 2.13	.037
Children vs adults				*t(72)*= 0.286	.629
Adolescents vs adults				*t(77.2)*= 2.00	.049
Days between sessions	4.1 (2.8)	3.8 (2.2)	4.5 (4.5)	*F*(2106)= 0.43	.653
Range	1–13	1–12	1–31		
Retention test time of day^b^	731 (136)	725 (153)	694 (128)	*F*(2103)= 0.722	.488
Children vs adolescents				*t(58.1)*= 0.17	.867
Children vs adults				*t(51)*= 1.11	.274
Adolescents vs adults				*t(74.1)*= 0.97	.335
US sound level % of max	10.9 (3.8)	41.7 (10.2)	49.6 (8.0)	*F*(2112)= 241.8	< .001
Range	10–30	15–70	30–60		
Children vs adolescents				*t(49.5)*= 17.5	< .001
Children vs adults				*t(61.1)*= 27.8	< .001
Adolescents vs adults				*t(71.9)*= 3.89	< .001
Contingency aware n (%)	26 (79 %)	34 (85 %)	41 (95 %)	χ^2^(2) = 4.78	.092

**Notes.** a: Socioeconomic status for parents of children and adolescents and for adult participants, educational level and yearly income, from 0 to 9. b. Number of minutes since midnight. US: unconditioned stimulus. *p < .05, ** p < .01, ***p < .001.

### 3.2 Skin conductance responses

Of the 120 participants included in the study, 3 (2 children and 1 adult) were excluded due to technical problems during SCR acquisition. This left data from 117 participants; 34 children (20 female, 14 male), 40 adolescents (21 female, 19 male), and 43 adults (22 female, 21 male). The groups had similar sex distributions (χ^2^(2) = 0.49, *p* = .78). Linear mixed-effects models (LMM) were used to analyze SCR during the different fear conditioning phases. We first performed whole-sample analyses looking at the main effect of stimulus and trial as well as their interaction. During the acquisition phase, there was a main effect of CS, with greater SCR to the CS+ than CS-, and trial, with smaller responses over time, as well as a CS × trial interaction indicating successful fear learning across groups (Table 2, Supplementary Figure 1). During the extinction learning and retention phases, there were main effects of CS and trial, as in the acquisition phase, but no CS × trial interactions.

**Table 2:** Linear mixed effects models of skin conductance responses for the fear conditioning phases. Main effects of conditioned stimulus (CS) and trial and their interactions. CS- reference condition.

Phase	β	SE	t	p
**Acquisition**				
CS: CS+	0.01	0.03	0.387	.699
trial	−0.01	0.003	2.596	.009 * *
CS × trial	0.01	0.005	3.307	< .001 * **
**Extinction learning**				
CS: CS+	0.06	0.02	2.579	.001 * *
trial	−0.01	0.003	5.241	< .001 * **
CS × trial	0.005	0.004	1.217	.223
**Retention test**				
CS: CS+	0.09	0.03	3.397	< .001 * **
trial	−0.02	0.003	4.878	< .001 * **
CS × trial	−0.01	0.004	1.432	.152

**Notes.** β = estimate. SE: Standard error. * p < .05, * * p < .01, * ** p < .001.

**Fig. 3:**
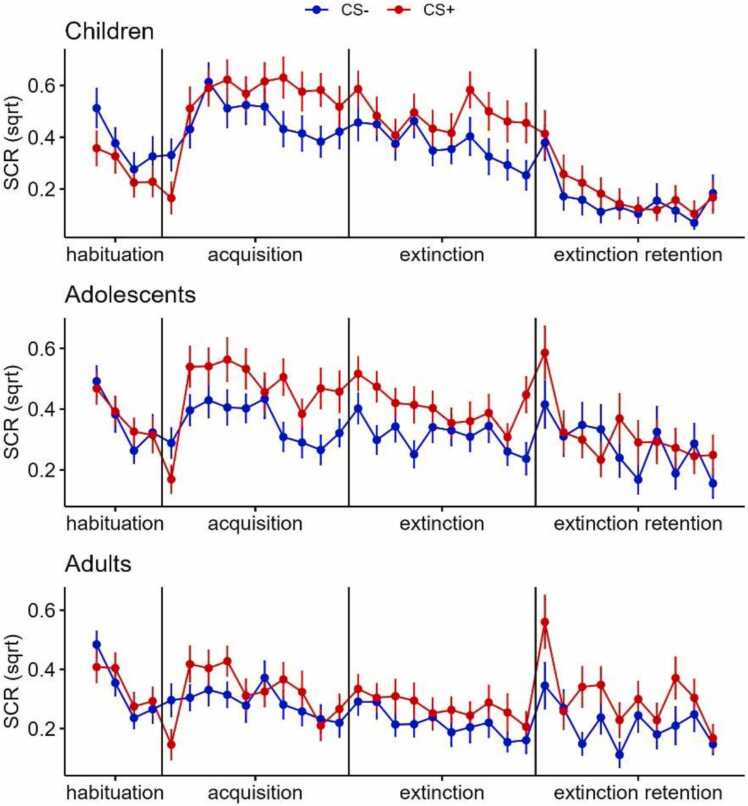
Square-root (sqrt) of skin conductance responses (SCR) to the conditioned stimulus paired with a scream (CS+) and never-paired (CS-) during the four phases of conditioning in children, adolescents, and adults. Error bars denote standard error.

We then performed LMMs adding age group, with adolescents as the reference group, and its interaction with CS and trial, to test for the proposed dip in extinction learning and extinction retention during adolescence. The only group effect that emerged was a CS × trial × group interaction during extinction learning indicating that children and adolescents differed in their separation between CS+ and CS- over trials during extinction (β = −0.02, *SE* = 0.01, *p* = .036). However, adolescents did not differ in SCR compared to adults during extinction learning or compared to children or adults during acquisition or extinction retention. We subsequently completed LMM analyses using children as a reference group, to test for progressive changes in fear conditioning processes across development. During fear acquisition and extinction learning, main effects of group (acquisition: β = −0.14, *SE* = 0.07, *p* = .042; extinction: β = 0.19, *SE* = 0.07, *p* = .003) showed that children had generally higher SCR than adults during these two phases, and a CS × trial × group interaction during the fear acquisition phase (β = −0.02, *SE* = 0.01, *p* = .041) indicated that during fear acquisition, adults differentiated less between CS+ and CS- over trials than children. The pattern of SCR is consistent with faster fear acquisition and declining fear response across trials (Fig. 3). We could not detect any CS × trial ×group interaction during extinction learning or extinction retention, suggesting similar extinction learning and retention from childhood to adulthood. As the fear response can be very brief during the extinction retention phase, we also calculated the difference in SCR between the first two CS+ and the first two CS- presentations during the retention phase and tested for group differences in this variable using a linear regression model. No effect of group was detected (*p’s* > .434), highlighting that all groups displayed similar extinction retention. See Fig. 3 for an illustration of SCR in all phases of the experiment.

US habituation was evident in the whole sample, with no group differences or group × trial interactions (Supplementary Table 1, Supplementary Figure 2).

### 3.3 Affective ratings

**Fig. 4:**
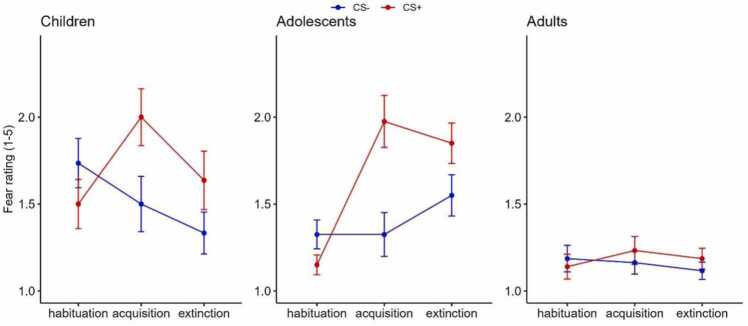
Fear ratings to conditioned stimuli. Ratings (1−5) of fear to the CS+ (red) and CS- (blue) before habituation, after fear acquisition, and after extinction learning in all age groups.

Affective ratings were analyzed using cumulative link mixed models. An initial model in the whole sample with CS and phase as predictors showed a main effect of CS, such that participants rated more fear to the CS+ than CS- (Supplementary Table 2). The difference between CS+ and CS- ratings was also greater after fear acquisition than after habituation as seen in the CS × phase interaction (β = 2.81, *SE* = 0.51, *p* < .001), indicating successful fear learning (Supplementary Figure 3). Adding age group, with adolescents as the reference group, to the model showed that adolescents rated more fear to the CS+ (>CS-) than adults over the course of all phases (β = 2.13, *SE* = 0.89, *p* = .020), with no differences between adolescents and children (Fig. 4). Adolescents also increased their fear response to the CS+ more during acquisition than adults (β = 2.85, *SE* = 1.34, *p* = .034), but not compared to children. Finally, adolescents reduced their fear ratings less to both the CS+ and the CS- than children during extinction learning (β = −1.90, *SE* = 0.87, *p* = .029), with Fig. 4 showing the pattern that adolescents only slightly reduced their fear ratings to CS+ and actually increased their fear ratings to CS-. Adding children as the reference group in the CLMM analysis yielded no additional group differences, indicating no group differences between children and adults. {{{Table 3}}}

**Table 3:** Cumulative link mixed effects models of fear ratings of the conditioned stimulus paired with the scream (CS+) and never-paired (CS-) pre habituation, after fear acquisition, and after extinction learning. CS- and acquisition reference conditions.

	β	SE	Z	p
CS: CS+	1.91	0.35	5.521	< .001 * **
Phase: pre	0.57	0.35	1.610	.107
Phase: extinction	0.18	0.36	0.504	.641
CS × phase: pre	−2.81	0.51	5.512	< .001 * **
CS × phase: extinction	−0.78	0.47	1.652	.099

**Notes.** β = estimate. SE: Standard error. * p < .05, * * p < .01, * ** p < .001.

### 3.4 Neural activity and connectivity during fear extinction retention

**Fig. 5:**
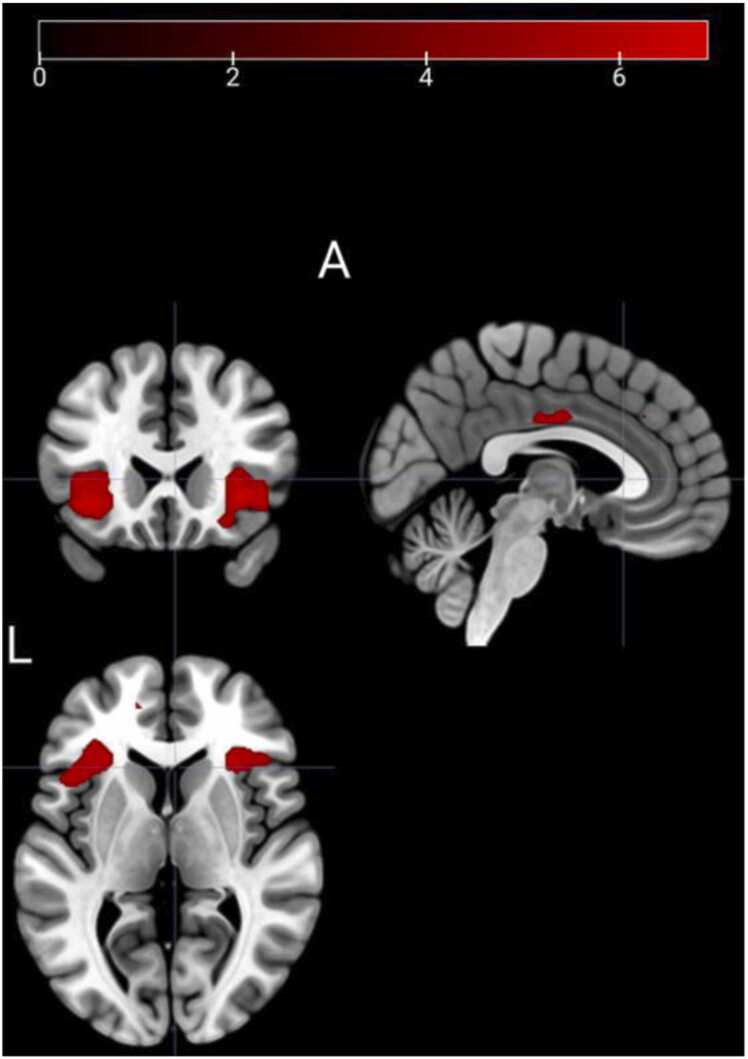
Fear retention in the brain. Across age groups, fear retention (CS+>CS-) was detected mainly in the insula and the mid-cingulate region, but also included clusters in the anterior PFC, and the paracingulate gyrus. Statistical parametric maps thresholded at family-wise error corrected (FWE) p_FWE_< .05 and overlaid on T1-weighted anatomical reference images. Color bar indicates Z scores. A: anterior, L: left.

Out of the 120 participants, 16 were excluded due to technical problems during fMRI acquisition, which left fMRI data from 104 participants: 26 children (16 female, 10 male), 37 adolescents (20 female, 17 male), and 41 adults (21 female, 20 male). The age groups had similar sex distributions (χ^2^(2) = 0.70, *p* = .71). The number of volumes censored at the 0.9 mm FD threshold differed between groups (*F*(2101)= 9.389, *p* < .001), and was highest in children (*M* (*SD*) 13 (21.4)), in the middle in adolescents (4.8 (7.3)), and lowest in adults (0.4 (1.5)). When all age groups were pooled together in whole-brain analyses, the CS+ >CS- contrast revealed activations in several brain areas, mainly the bilateral insular cortex and ACC (see Table 4, Fig. 5), indicative of fear retention. When comparing the groups, we only detected one group difference. Adolescents had higher activity in the right amygdala (ROI analysis) than children for the CS+ >CS- contrast (peak MNI coordinates: 18, 0, −14; Z = 3.47; *p*_FWE_ = 0.023; cluster volume:16 mm^3^). No other group differences were detected in whole-brain or in the amygdala, insula, hippocampus, ACC or vmPFC ROI analyses. No CS+ >CS- seed-to-voxel functional connectivity (gPPI) in the whole sample, or age group differences in such connectivity, were detected for any of our pre-defined seeds; the amygdala, insula, hippocampus, ACC and vmPFC.

**Table 4:** Blood-oxygenation-level dependent responses to the CS+ >CS- contrast during the retention test. Whole-brain analyses thresholded at family-wise error corrected (FWE) p_FWE_< .05.

	Hemisphere	MNI x, y, z			Z	*p*_FWE_	cluster volume^a^
Insular cortex	Left	−38	20	−6	6.30	< .001	5080
Frontal orbital cortex / insular cortex	Right	32	26	−4	6.11	< .001	5400
Anterior cingulate cortex	Bilateral	2	−6	34	5.30	.002	1280
Anterior cingulate cortex	Left	−14	26	26	5.10	.006	136
Paracingulate gyrus	Left	−14	50	6	4.76	.026	32
Middle temporal gyrus	Right	52	−20	−6	4.63	.045	16
Paracingulate gyrus	Right	2	32	34	4.61	.047	16

MNI: Montreal Neurological Institute. Brain regions from the Harvard-Oxford atlas.

^a^Cluster volume in mm^3^

^b^Only the detected group differences reported in the table.

### 3.5 Sensitivity analyses

We performed a series of sensitivity analyses for the SCR and brain activity results, with none of these analyses altering the pattern of results. First, to account for group difference in SES, we added SES as a covariate to the analyses. These analyses did not change the pattern of results (Supplementary Table 2 and 3). To account for group difference in US sound level, we performed sensitivity analyses adding this as a covariate, which did not alter the pattern of results (Supplementary Table 4 and 5). We then performed sensitivity analyses of extinction retention, accounting for the number of days between session 1 and 2, which also produced similar results (Supplementary Table 6 and 7). Finally, we performed sensitivity analyses including only the participants who were CS-US contingency aware, which produced similar results as including all participants (Supplementary Table 8 and 9).

## 4 Discussion

We used Pavlovian fear conditioning in children, adolescents, and adults to test, for the first time in humans, the proposed adolescent deficits in extinction learning and retention using affective ratings as well as physiological and neural measures. (Kitt et al., 2023, Lau et al., 2011, Pattwell et al., 2012). We found no evidence of a robust dip in extinction learning or extinction retention in adolescence in physiological, self-report, or neural correlates of fear conditioning. There were no group differences in differential SCR to the CS+ >CS- during extinction learning or extinction retention, all age groups instead showing similar extinction learning and extinction retention patterns. The findings during extinction learning are in line with the largest study to date by Abend et al., (2020), who did not find any relationship between age and extinction learning, but in contrast to the study by Pattwell et al. (2012), who initially reported the adolescent extinction dip in humans. Affective ratings of fear to the CS+ and the CS- differed somewhat between age groups, but we could not find a stronger fear response (CS+>CS-) specifically after extinction learning in adolescents compared to children and adults. This is in contrast to Waters et al. (2017), who reported less positive re-evaluations of CS during extinction learning in adolescents compared to children and adults that also remained to the extinction retention test. We found greater fear response (CS+>CS-) in the right amygdala during the retention test in adolescents compared to children, but not compared to adults, and there were no other age-dependent differences in brain fear response in any other brain area. Further, we did not detect any developmental changes in amygdala-PFC connectivity. Instead, pooling all age groups together yielded greater neural activity to the CS+ >CS- contrast during the retention test, prominently in the insula and anterior cingulate cortex, suggesting similar neural activation patterns across development. Hence, our results do not support the existence of diminished fear extinction learning or extinction retention in adolescence compared to childhood and adulthood (Kitt et al., 2023, Lau et al., 2011, Pattwell et al., 2012), suggesting that there is not a robust dip in fear extinction during adolescence.

In addition to the test of an adolescent extinction dip, we also tested for progressive changes in fear conditioning processes across development. As reported above, we found little evidence for a progressive change in extinction learning or extinction retention from childhood to adulthood. However, we did detect changes in fear acquisition between children and adults using SCR, with adults acquiring the fear response on earlier trials; i.e., they differentiated faster between the CS+ and CS-, and then their fear response declined more rapidly. This is in accordance with previous work showing that learning to differentiate between threat and safety cues develops during childhood (Gao et al., 2010). Hence, our findings indicate only minor differences in how children, adolescents, and adults learn, extinguish, and retain fear responses. Instead, all three age groups seem to acquire and extinguish fear, as measured by SCR and affective ratings, with shared neural fear response (CS+>CS-) in the insula and ACC during the extinction retention test. Overall, our results support the view that fear conditioning generally elicits similar physiological, subjective, and neural responses across different stages of development from early school age to mid-adulthood (Stenson et al., 2023).

Some methodological considerations are of importance in the interpretation of the results. Return of extinguished fear is thought to involve three main processes, namely reinstatement, renewal, and spontaneous recovery (Bouton, 2004). Reinstatement occurs when fear returns following reencountering the US (or another stressor) after extinction, renewal refers to fear returning when the CS+ is encountered in another context than the one where extinction took place, and spontaneous recovery refers to when fear returns following the passage of time. In a clinical setting, all of these processes are important in explaining why fear returns in patients following successful extinction training. In a research context, many opt to study each of these processes in isolation so as to avoid confounding effects from the other factors. In the present study, the context in which acquisition and extinction learning took place was different from the context in which the retention test took place, and at least 24 h had passed since extinction. Therefore, both renewal and spontaneous recovery are likely to have influenced fear extinction retention in the participants.

Another important aspect is the time between fear acquisition and extinction learning, and this may be an important moderator of the extinction dip seen in some studies and not others. Most studies to date have performed extinction training immediately following or after a short interval (<1 h) (Stenson et al., 2023). The only study with a longer interval (>24 h) was the study by Pattwell and colleagues (Pattwell et al., 2012), which was the first to report the dip in adolescent fear extinction. In the present study, there was a 1.5 h interval between fear acquisition and extinction as the participants performed different cognitive tasks and had a break of about 15 min between these phases. Similar to studies using the shorter intervals, we also found age-invariant fear acquisition and extinction learning, potentially pointing to timing as a crucial factor in understanding the mixed findings. The longer time utilized by Pattwell and colleagues enables consolidation of fear memories, whereas the shorter time used in this study and others do not allow for full memory consolidation. Thus, fear extinction may depend on different processes depending on the experimental setup. Hence, timing between fear conditioning phases should be more systematically addressed in future studies.

For the fMRI analyses, when data from all of the participants was pooled, we found fear-memory associated (CS+>CS-) activation in the bilateral insula and the ACC during the retention test. This is in accordance with the meta-analysis by Fullana et al. (2018) who also found consistent activation in the insula and the ACC in both extinction learning and retention studies. Both the insula and the ACC have been linked to threat appraisal and the experience of threats, so greater activity in these regions to the CS+ (compared to CS-) in this study adheres to previous research linking these regions to threat appraisal. The only age-related difference in brain activity was that adolescents had a greater right amygdala fear response than children, but not adults. While this lends some support to adolescence being a period of attenuated extinction retention compared to children, no differences in amygdala activation were found compared to adults, and no other differences in brain activity or connectivity to the CS+ >CS- contrast were detected. Overall, our findings are in accordance with the idea that threat processing in the brain is generally similar from childhood to adulthood.

The fear conditioning paradigm used in the current study, the Screaming lady task, has previously been questioned for its suitability in fear conditioning studies involving children and adolescents due to it possibly being too aversive (Shechner et al., 2015a, Shechner et al., 2015b), bearing the risk of children and adolescents withdrawing from studies. In the current study, 13 out of 148 participants discontinued participation due to the paradigm being too aversive. Twelve of these participants were children and one was an adolescent, indicating that it was indeed more aversive for children than the other age groups. Most of these withdrawals occurred during the initial phase of the study. Contributing to this, children often misjudged what volume would be ‘aversive, but tolerable’ to them when they were allowed to select the volume themselves. We found that setting the volume for children at a pre-determined level resulted in fewer drop-outs. After implementing this adjustment, we had very few withdrawals from the study. While great care is needed when conducting research with aversive stimuli, the aversiveness also can address possible criticisms. For example, one could attribute the absence of group differences to the less aversive nature of a scream than the types of more aversive USs employed with adults. However, given the aversiveness of the scream in this study, it would be hard to justify a more aversive stimulus. Nonetheless, to the extent that it is possible to conduct ethical research with aversive stimuli, our results suggest that all age groups show similar levels of fear acquisition and extinction based on SCR and self-report, although the timing of the CS differentiation may vary between children and adults. Therefore, our findings indicate that the paradigm can be successfully used in fear conditioning experiments across development.

This study has some limitations that deserve notice. First, fear acquisition and extinction learning were performed in a laboratory whereas the retention test was performed in the MR scanner. This context shift could result in less robust extinction retention, as extinction may not generalize to a new context. However, if there was such an effect, it did not result in any group differences in extinction retention. As noted above, multiple processes may have contributed to the responses to CS+ and CS- during the retention test, including renewal and spontaneous recovery. We cannot differentiate these processes using the current design, which is a limitation of the study. Thus, we cannot rule out that age group differences in these two processes may have been in opposite directions and counteracted each other, producing the reported null results. Another potential limitation is that we did not use an unextinguished CS+ in the retention test. This has been discussed as a potential limitation in fear extinction studies (Fullana et al., 2018), as the extinguished CS+ is proposed to potentially signal both safety and fear through the inhibitory learning thought to take place during fear extinction (Craske et al., 2014). Thus, including a non-extinguished CS+ could potentially elicit a stronger fear response at a retention test. We did not include subjective affective ratings of CS+ and CS- at the beginning of the retention test, which would have added important information regarding extinction retention. Also, we did not have access to common measures of anxiety and other mental health symptoms across the age groups, which is a limitation as potential group differences in e.g. anxiety levels may confound the results. We did find group differences in US volume employed as well as SES indicators, but sensitivity analyses adding these variables as covariates did not alter the patten of results. Additionally, the relatively small sample size could limit the chance of finding differences between groups. Yet, this is one of the larger studies to date in the field, and studies that have found between-group effects previously have generally used similar, or smaller, samples. The generalizability of findings may also be reduced by the aversive nature of the fear conditioning task, specifically resulting in a larger proportion of children not completing the study. Moreover, the current study was cross-sectional and thus did not follow participants longitudinally. To further investigate fear conditioning and potential developmental differences, longitudinal studies should be utilized.

In conclusion, we found no evidence of attenuated extinction learning or extinction retention specific to adolescence, and no major alterations in extinction learning or extinction retention from childhood to adulthood. Our results instead indicate consistent fear acquisition and extinction across development and suggest that the reported dip in fear extinction in adolescence is not a robust phenomenon.

## CRediT authorship contribution statement

**Daniel S. Pine:** Writing – review & editing, Supervision, Resources, Methodology. **Karin Brocki:** Writing – review & editing, Supervision, Resources. **David Fällmar:** Writing – review & editing, Investigation. **Malin Gingnell:** Writing – review & editing, Supervision, Resources, Project administration, Methodology, Investigation, Funding acquisition, Conceptualization. **Ebba Widegren:** Writing – original draft, Visualization, Project administration, Methodology, Investigation, Formal analysis, Conceptualization. **Andreas Frick:** Writing – review & editing, Visualization, Supervision, Resources, Project administration, Investigation, Funding acquisition, Formal analysis, Data curation, Conceptualization. **Johan Vegelius:** Writing – review & editing, Supervision, Formal analysis. **Mathilda A. Frick:** Writing – review & editing, Methodology, Investigation. **Ashika Roy:** Writing – review & editing, Formal analysis. **Stefan Möller:** Writing – review & editing, Investigation. **Johan Lundin Kleberg:** Writing – review & editing, Software, Methodology. **Johanna M. Hoppe:** Writing – review & editing, Investigation. **Olof Hjorth:** Writing – review & editing, Investigation.

## Declaration of Competing Interest

The authors declare that they have no known competing financial interests or personal relationships that could have appeared to influence the work reported in this paper.

## Data Availability

Data will be made available on request.
