# Supplementary material for: Fear extinction retention in children, adolescents, and adults
Source: Dev Cogn Neurosci. 2025 Jan 9;71:101509. doi: 10.1016/j.dcn.2025.101509 (PMC11773086; doi:10.1016/j.dcn.2025.101509)
Supplement: Supplementary Figure 1 — Supplementary material [file mmc1.pdf]

**Fear extinction retention in children, adolescents, and adults**

**Supplemental Material**

Ebba Widegren[a], Johan Vegelius[a], Matilda A. Frick[b,c], Ashika A. Roy[a], Stefan Möller[d], Johan Lundin Kleberg[c], Johanna Motilla Hoppe[a,e], Olof Hjorth[e], David Fällmar[f], Daniel S. Pine[g], Karin Brocki[e], Malin Gingnell[a]§, Andreas Frick[a]§

[a]Department of Medical Sciences, Psychiatry, Uppsala University, Uppsala, Sweden
[b]Department of Medical Sciences, Child and Adolescent Psychiatry, Uppsala University, Uppsala, Sweden
[c]Department of Psychology, Stockholm University, Stockholm, Sweden
[d]Department of Psychology, Lund University, Lund, Sweden
[e]Department of Psychology, Uppsala University, Uppsala, Sweden
[f]Department of Surgical Sciences, Neuroradiology, Uppsala University Hospital, Uppsala, Sweden
[g]Section on Development and Affective Neuroscience, National Institute of Mental Health Intramural Research Program, Bethesda, MD, USA
§ Equal contribution

## METHODS

### Magnetic resonance imaging preprocessing

Results included in this manuscript come from preprocessing performed using *fMRIPrep* 23.1.4 (Esteban et al. (2019); Esteban et al. (2018); RRID:SCR_016216), which is based on *Nipype* 1.8.6 (K. Gorgolewski et al. (2011); K. J. Gorgolewski et al. (2018); RRID:SCR_002502).

#### *Anatomical data preprocessing*

A total of 1 T1-weighted (T1w) images were found within the input BIDS dataset. The T1-weighted (T1w) image was corrected for intensity non-uniformity (INU) with N4BiasFieldCorrection (Tustison et al. 2010), distributed with ANTs (version unknown) (Avants et al. 2008, RRID:SCR_004757), and used as T1w-reference throughout the workflow. The T1w-reference was then skull-stripped with a *Nipype* implementation of the antsBrainExtraction.sh workflow (from ANTs), using OASIS30ANTs as target template. Brain tissue segmentation of cerebrospinal fluid (CSF), white-matter (WM) and gray-matter (GM) was performed on the brain-extracted T1w using fast (FSL (version unknown), RRID:SCR_002823, Zhang, Brady, and Smith 2001). Brain surfaces were reconstructed using recon-all (FreeSurfer 7.3.2, RRID:SCR_001847, Dale, Fischl, and Sereno 1999), and the brain mask estimated previously was refined with a custom variation of the method to reconcile ANTs-derived and FreeSurfer-derived segmentations of the cortical gray-matter of Mindboggle (RRID:SCR_002438, Klein et al. 2017). Volume-based spatial normalization to one standard space (MNI152NLin2009cAsym) was performed through nonlinear registration with antsRegistration (ANTs (version unknown)), using brain-extracted versions of both T1w reference and the T1w template. The following template was were selected for spatial normalization and accessed with *TemplateFlow* (23.0.0, Ciric et al. 2022): *ICBM 152 Nonlinear Asymmetrical template version 2009c* [Fonov et al. (2009), RRID:SCR_008796; TemplateFlow ID: MNI152NLin2009cAsym].

#### *Functional data preprocessing*

For each of the 2 BOLD runs found per subject (across all tasks and sessions), the following preprocessing was performed. First, a reference volume and its skull-stripped version were generated using a custom methodology of *fMRIPrep*. Head-motion parameters with respect to the BOLD reference (transformation matrices, and six corresponding rotation and translation parameters) are estimated before any spatiotemporal filtering using mcflirt (FSL , Jenkinson et al. 2002). BOLD runs were slice-time corrected to 0.969s (0.5 of slice acquisition range 0s-1.94s) using 3dTshift from AFNI (Cox and Hyde 1997, RRID:SCR_005927). The BOLD time-series (including slice-timing correction when applied) were resampled onto their original, native space by applying the transforms to correct for head-motion. These resampled BOLD time-series will be referred to as *preprocessed BOLD in original space*, or just *preprocessed BOLD*. The BOLD reference was then co-registered to the T1w reference using bbregister (FreeSurfer) which implements boundary-based registration (Greve and Fischl 2009). Co-registration was configured with six degrees of freedom. Several confounding time-series were calculated based on the *preprocessed BOLD*: framewise displacement (FD), DVARS and three region-wise global signals. FD was computed using two formulations following Power (absolute sum of relative motions, Power et al. (2014)) and Jenkinson (relative root mean square displacement between affines, Jenkinson et al. (2002)). FD and DVARS are calculated for each functional run, both using their implementations in *Nipype* (following the definitions by Power et al. 2014). The three global signals are extracted within the CSF, the WM, and the whole-brain masks. Additionally, a set of physiological regressors were extracted to allow for component-based noise correction (*CompCor*, Behzadi et al.

2007). Principal components are estimated after high-pass filtering the *preprocessed BOLD* time-series (using a discrete cosine filter with 128s cut-off) for the two *CompCor* variants: temporal (tCompCor) and anatomical (aCompCor). tCompCor components are then calculated from the top 2% variable voxels within the brain mask. For aCompCor, three probabilistic masks (CSF, WM and combined CSF+WM) are generated in anatomical space. The implementation differs from that of Behzadi et al. in that instead of eroding the masks by 2 pixels on BOLD space, a mask of pixels that likely contain a volume fraction of GM is subtracted from the aCompCor masks. This mask is obtained by dilating a GM mask extracted from the FreeSurfer's *aseg* segmentation, and it ensures components are not extracted from voxels containing a minimal fraction of GM. Finally, these masks are resampled into BOLD space and binarized by thresholding at 0.99 (as in the original implementation). Components are also calculated separately within the WM and CSF masks. For each CompCor decomposition, the *k* components with the largest singular values are retained, such that the retained components' time series are sufficient to explain 50 percent of variance across the nuisance mask (CSF, WM, combined, or temporal). The remaining components are dropped from consideration. The head-motion estimates calculated in the correction step were also placed within the corresponding confounds file. The confound time series derived from head motion estimates and global signals were expanded with the inclusion of temporal derivatives and quadratic terms for each (Satterthwaite et al. 2013). Frames that exceeded a threshold of 0.5 mm FD or 1.5 standardized DVARS were annotated as motion outliers. We here used the FD threshold of 0.9 mm for censoring volumes. Additional nuisance timeseries are calculated by means of principal components analysis of the signal found within a thin band (*crown*) of voxels around the edge of the brain, as proposed by (Patriat, Reynolds, and Birn 2017). The BOLD time-series were resampled into standard space, generating a *preprocessed BOLD run in MNI152NLin2009cAsym space*. First, a reference volume and its skull-stripped version were generated using a custom methodology of *fMRIPrep*. The BOLD time-series were resampled onto the following surfaces (FreeSurfer reconstruction nomenclature): *fsnative*, *fsaverage5*. All resamplings can be performed with *a single interpolation step* by composing all the pertinent transformations (i.e. head-motion transform matrices, susceptibility distortion correction when available, and co-registrations to anatomical and output spaces). Gridded (volumetric) resamplings were performed using antsApplyTransforms (ANTs), configured with Lanczos interpolation to minimize the smoothing effects of other kernels (Lanczos 1964). Non-gridded (surface) resamplings were performed using mri_vol2surf (FreeSurfer). Many internal operations of *fMRIPrep* use *Nilearn* 0.10.1 (Abraham et al. 2014, RRID:SCR_001362), mostly within the functional processing workflow. For more details of the pipeline, see the section corresponding to workflows in *fMRIPrep*'s documentation.

## RESULTS

***Supplementary Table 1.*** *Linear mixed effects model of skin conductance responses to the unconditioned stimulus scream. Main effects of trial and group and their interactions.*

| | **β** | **SE** | **t** | **p** |
|---|---|---|---|---|
| Trial | -0.07 | 0.06 | 9.532 | <.001 *** |
| Group: 1 vs 2 | -0.02 | 0.01 | 0.240 | .810 |
| Group: 1 vs 3 | 0.11 | 0.09 | 1.251 | .213 |
| Group: 2 vs 3 | 0.13 | 0.08 | 1.552 | .122 |
| Trial × Group: 1 vs 2 | -0.01 | 0.09 | 0.748 | .455 |
| Trial × Group: 1 vs 3 | -0.02 | 0.01 | 1.877 | .061 |
| Trial × Group: 2 vs 3 | -0.01 | 0.01 | 1.167 | .244 |

**Notes.** β = estimate. SE: Standard error. * p < .05, ** p < .01, *** p < .001.

***Supplementary Table 2.*** *Linear mixed effects models of skin conductance responses for the fear conditioning phases while accounting for socioeconomic status (SES) of parents (children and adolescents) or participants (adults). Main effects of conditioned stimulus (CS), trial and their interactions.*

| **Phase** | **β** | **SE** | **t** | **p** |
|---|---|---|---|---|
| **Acquisition** | | | | |
| CS: CS+ | 0.01 | 0.03 | 0.444 | .657 |
| trial | -0.01 | 0.003 | 2.791 | **.005** ** |
| CS × trial | 0.02 | 0.005 | 3.397 | **<.001** *** |
| SES | -0.03 | 0.01 | 1.842 | **.068** ** |
| **Extinction learning** | | | | |
| CS: CS+ | 0.06 | 0.02 | 2.452 | **.014** ** |
| Trial | -0.01 | 0.003 | 5.165 | **<.001** *** |
| CS × trial | 0.005 | 0.004 | 1.256 | .209 |
| SES | 0.04 | 0.01 | 3.083 | **.003** ** |
| **Retention test** | | | | |
| CS: CS+ | 0.09 | 0.03 | 3.050 | **<.001** *** |
| Trial | -0.02 | 0.003 | 5.007 | **<.001** *** |
| CS × trial | -0.01 | 0.004 | 1.118 | .264 |
| SES | 0.006 | 0.02 | 0.396 | .693 |

**Notes.** β = estimate. SE: Standard error. * p < .05, ** p < .01, *** p < .001.

***Supplementary Table 3.*** *Brain activation to the CS+>CS- contrast during the retention test, while accounting for socioeconomic status. Statistical threshold family-wise error corrected (FWE) $p_{FWE}$<.05.*

| **Brain area** | **Hemisphere** | **MNI x, y, z** | | | **Z** | $p_{\text{FWE}}$ | **volume**[a] |
|---|---|---|---|---|---|---|---|
| Insular cortex | Left | -38 | 20 | -6 | 6.08 | <.001 | 4208 |
| Frontal orbital cortex / insular cortex | Right | 32 | 26 | -4 | 5.88 | <.001 | 4704 |
| Anterior cingulate cortex | Bilateral | 2 | -6 | 34 | 5.02 | .008 | 648 |
| Anterior cingulate cortex | Left | -14 | 26 | 26 | 4.96 | .011 | 88 |
| Paracingulate gyrus | Left | -14 | 50 | 8 | 4.65 | .040 | 8 |

MNI: Montreal Neurological Institute

[a] Cluster volume in $mm^3$

***Supplementary Table 4.*** *Linear mixed effects models of skin conductance responses for the fear conditioning phases while accounting for sound level of the unconditioned stimulus (US). Main effects of conditioned stimulus (CS), trial and their interactions.*

| **Phase** | **β** | **SE** | **t** | **p** |
|---|---|---|---|---|
| **Acquisition** | | | | |
| CS: CS+ | 0.01 | 0.03 | 0.401 | .688 |
| trial | -0.01 | 0.003 | 2.455 | **.014** * |
| CS × trial | 0.02 | 0.005 | 3.334 | **<.001** *** |
| Sound level | -0.003 | 0.001 | 2.925 | **.004** ** |
| **Extinction learning** | | | | |
| CS: CS+ | 0.06 | 0.02 | 2.623 | **.009** ** |
| Trial | -0.01 | 0.003 | 5.045 | **<.001** *** |
| CS × trial | 0.004 | 0.004 | 1.108 | .268 |
| Sound level | -0.003 | 0.001 | 2.427 | **.017** * |
| **Retention test** | | | | |
| CS: CS+ | 0.09 | 0.03 | 3.335 | **<.001** *** |
| Trial | -0.02 | 0.003 | 4.825 | **<.001** *** |
| CS × trial | -0.01 | 0.004 | 1.432 | .137 |
| Sound level | 0.003 | 0.001 | 2.187 | **.031** * |

**Notes.** β = estimate. SE: Standard error. * p < .05, ** p < .01, *** p < .001.

***Supplementary Table 5.*** *Brain activation to the CS+>CS- contrast during the retention test, while accounting for sound level of the unconditioned stimulus (US). Statistical threshold family-wise error corrected (FWE)* $p_{FWE}$*<.05.*

| Brain area | Hemisphere | MNI x, y, z | | | Z | $p_{FWE}$ | volume[a] |
|---|---|---|---|---|---|---|---|
| Anterior cingulate cortex | Bilateral | 2 | -6 | 32 | 5.72 | <.001 | 1216 |
| Insular cortex | Right | 32 | 26 | 0 | 5.49 | .001 | 2184 |
| Insular cortex | Bilateral | -38 | 20 | -6 | 5.32 | .002 | 2016 |
| Frontal orbital cortex | Left | 26 | 20 | -14 | 4.73 | .027 | 32 |
| Anterior cingulate cortex | Left | -14 | 26 | 26 | 4.63 | .041 | 32 |

MNI: Montreal Neurological Institute

[a] Cluster volume in $mm^3$

***Supplementary Table 6.*** *Linear mixed effects models of skin conductance responses for the retention test while accounting for days between sessions. Main effects of conditioned stimulus (CS), trial and their interactions. CS- reference condition.*

| | β | SE | t | p |
|---|---|---|---|---|
| **Retention test** | | | | |
| CS: CS+ | 0.09 | 0.03 | 3.397 | **<.001** *** |
| Trial | -0.02 | 0.003 | 4.878 | **<.001** *** |
| CS × trial | -0.01 | 0.004 | 1.432 | .152 |
| Days between sessions | 0.02 | 0.007 | 2.202 | **.030** * |

**Notes.** β = estimate. SE: Standard error. * p < .05, ** p < .01, *** p < .001.

***Supplementary Table 7.*** *Brain activation to the CS+>CS- contrast during the retention test, while accounting for days between sessions. Statistical threshold family-wise error corrected (FWE)* $p_{FWE}$*<.05.*

| Brain area | Hemisphere | MNI x, y, z | | | Z | $p_{FWE}$ | volume[a] |
|---|---|---|---|---|---|---|---|
| Insular cortex | Left | -38 | 20 | -6 | 6.28 | <.001 | 5240 |
| Frontal orbital cortex / insular cortex | Right | 32 | 26 | -4 | 6.11 | <.001 | 5400 |
| Anterior cingulate cortex | Bilateral | 2 | -6 | 34 | 5.27 | .003 | 1224 |
| Anterior cingulate cortex | Left | -14 | 28 | 26 | 5.13 | .005 | 160 |
| Paracingulate gyrus | Left | -14 | 50 | 6 | 4.74 | .028 | 32 |
| Paracingulate gyrus | Right | 2 | 34 | 34 | 4.70 | .033 | 56 |
| Middle temporal gyrus | Right | 52 | -20 | -6 | 4.62 | .045 | 16 |

MNI: Montreal Neurological Institute

[a] Cluster volume in $mm^3$

***Supplementary Table 8.*** *Linear mixed effects models of skin conductance responses for the fear conditioning phases in contingency aware participants only. Main effects of conditioned stimulus (CS), trial and their interactions.*

| Phase | β | SE | t | p |
|---|---|---|---|---|
| **Acquisition** | | | | |
| CS: CS+ | 0.01 | 0.03 | 0.391 | .696 |
| trial | -0.01 | 0.003 | 2.230 | **.026** * |
| CS × trial | 0.01 | 0.005 | 2.776 | **.006** ** |
| **Extinction learning** | | | | |
| CS: CS+ | 0.07 | 0.03 | 2.777 | **.006** ** |
| Trial | -0.01 | 0.003 | 4.933 | **<.001** *** |
| CS × trial | 0.003 | 0.004 | 0.679 | .497 |
| **Retention test** | | | | |
| CS: CS+ | 0.10 | 0.03 | 3.480 | **<.001** *** |
| Trial | -0.01 | 0.003 | 4.369 | **<.001** *** |
| CS × trial | -0.01 | 0.004 | 1.680 | .093 |

**Notes.** β = estimate. SE: Standard error. * $p < .05$, ** $p < .01$, *** $p < .001$.

***Supplementary Table 9.*** *Brain activation to the CS+>CS- contrast during the retention test in contingency aware participants only. Statistical threshold family-wise error corrected (FWE)* $p_{FWE}<.05$.

| Brain area | Hemisphere | MNI x, y, z | | | Z | $p_{FWE}$ | volume[a] |
|---|---|---|---|---|---|---|---|
| Insular cortex | Left | -38 | 20 | -6 | 5.78 | <.001 | 3112 |
| Insular cortex | Right | 38 | 32 | -2 | 5.50 | .001 | 3192 |
| Anterior cingulate cortex | Bilateral | 0 | -4 | 34 | 4.70 | .030 | 48 |
| Anterior cingulate cortex | Left | -14 | 28 | 26 | 4.59 | .048 | 16 |

MNI: Montreal Neurological Institute

[a] Cluster volume in $mm^3$

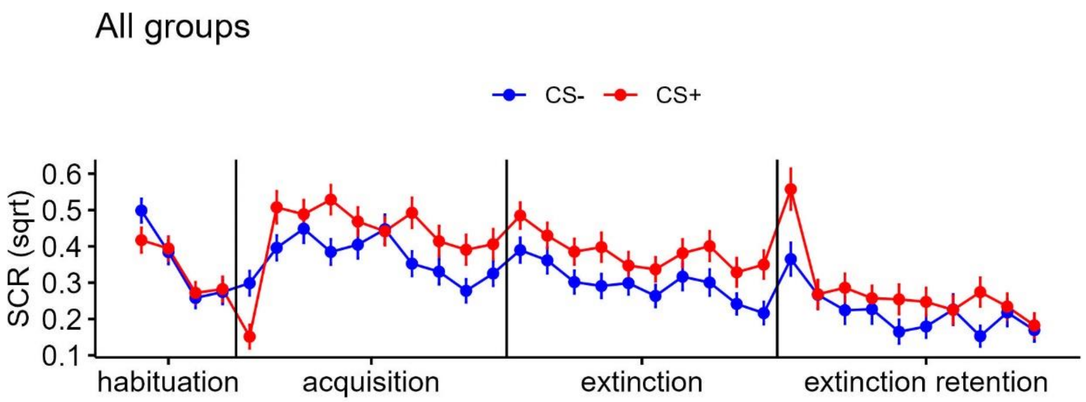


**Supplementary Figure 1**. Square-root (sqrt) of skin conductance responses (SCR) to the conditioned stimulus paired with a scream (CS+) and never-paired (CS-) during the four phases of conditioning in the whole sample. Error bars denote standard error.

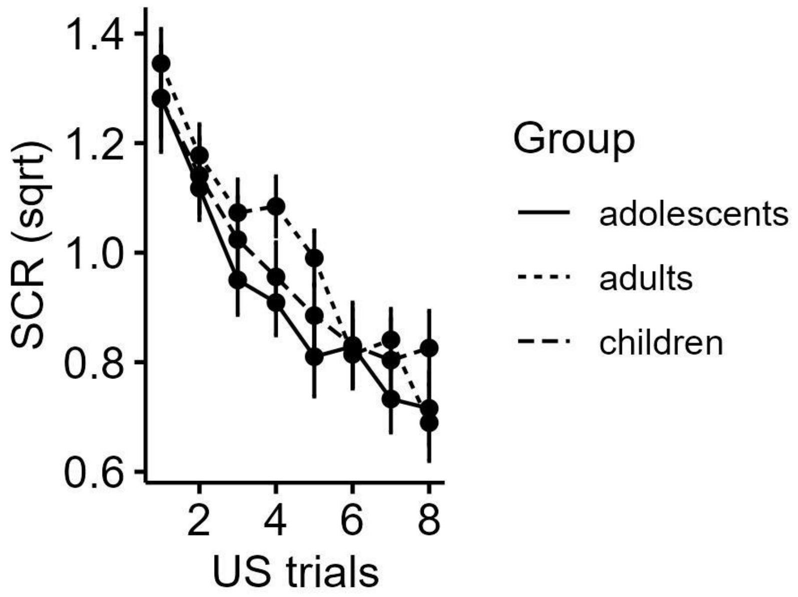


**Supplementary Figure 2**. Square root (sqrt) of skin conductance responses (SCR) to the unconditioned stimulus (US) scream for children, adolescents, and adults. Similar levels of SCR and habituation across trials were noted in all age groups. Error bars denote standard error.

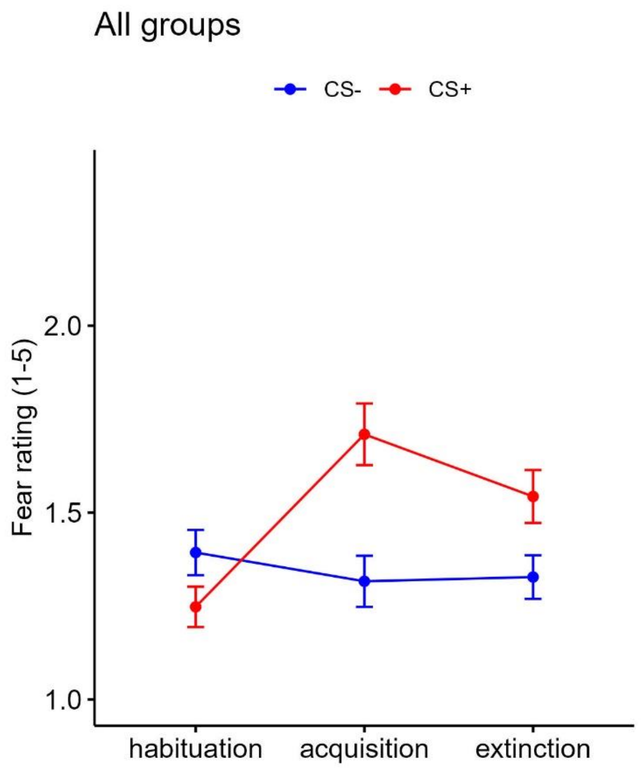


**Supplementary Figure 3**. **Fear ratings to conditioned stimuli.** *Ratings (1-5) of fear to the CS+ (red) and CS- (blue) before habituation, after fear acquisition, and after extinction learning in the whole sample*

.

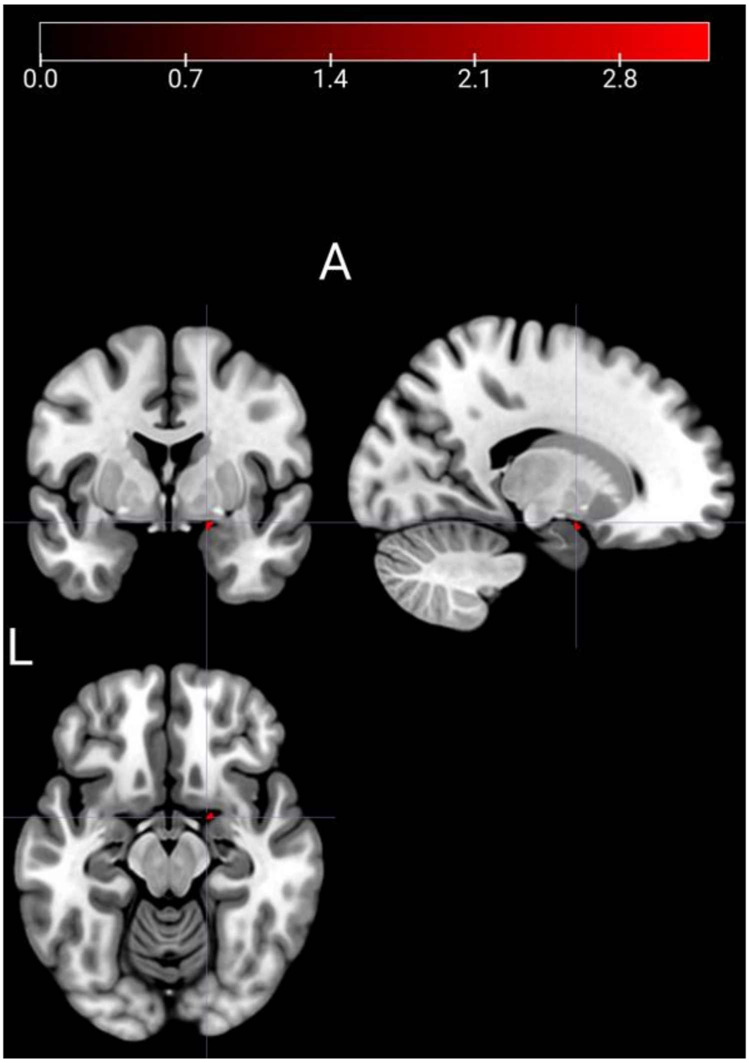


**Supplementary Figure 4.** ***Group difference between adolescents and children.*** *Attenuated extinction retention (CS+>CS-) was observed in the right amygdala in adolescents compared to children. Statistical parametric maps thresholded at p<.05 family-wise corrected within the amygdala region of interest overlaid on T1-weighted anatomical reference images. Color bar indicates Z scores. A: anterior, L: left.*

## REFERENCES


Abraham, Alexandre, Fabian Pedregosa, Michael Eickenberg, Philippe Gervais, Andreas Mueller, Jean Kossaifi, Alexandre Gramfort, Bertrand Thirion, and Gael Varoquaux. 2014. "Machine Learning for Neuroimaging with Scikit-Learn." *Frontiers in Neuroinformatics* 8. https://doi.org/10.3389/fninf.2014.00014.

Avants, B. B., C. L. Epstein, M. Grossman, and J. C. Gee. 2008. "Symmetric Diffeomorphic Image Registration with Cross-Correlation: Evaluating Automated Labeling of Elderly and Neurodegenerative Brain." *Medical Image Analysis* 12 (1): 26–41. https://doi.org/10.1016/j.media.2007.06.004.

Behzadi, Yashar, Khaled Restom, Joy Liau, and Thomas T. Liu. 2007. "A Component Based Noise Correction Method (CompCor) for BOLD and Perfusion Based fMRI." *NeuroImage* 37 (1): 90–101. https://doi.org/10.1016/j.neuroimage.2007.04.042.

Ciric, R., William H. Thompson, R. Lorenz, M. Goncalves, E. MacNicol, C. J. Markiewicz, Y. O. Halchenko, et al. 2022. "TemplateFlow: FAIR-Sharing of Multi-Scale, Multi-Species Brain Models." *Nature Methods* 19: 1568–71. https://doi.org/10.1038/s41592-022-01681-2.

Cox, Robert W., and James S. Hyde. 1997. "Software Tools for Analysis and Visualization of fMRI Data." *NMR in Biomedicine* 10 (4-5): 171–78. https://doi.org/10.1002/(SICI)1099-1492(199706/08)10:4/5<171::AID-NBM453>3.0.CO;2-L.

Dale, Anders M., Bruce Fischl, and Martin I. Sereno. 1999. "Cortical Surface-Based Analysis: I. Segmentation and Surface Reconstruction." *NeuroImage* 9 (2): 179–94. https://doi.org/10.1006/nimg.1998.0395.

Esteban, Oscar, Ross Blair, Christopher J. Markiewicz, Shoshana L. Berleant, Craig Moodie, Feilong Ma, Ayse Ilkay Isik, et al. 2018. "fMRIPrep 23.1.4." *Software*. https://doi.org/10.5281/zenodo.852659.

Esteban, Oscar, Christopher Markiewicz, Ross W Blair, Craig Moodie, Ayse Ilkay Isik, Asier Erramuzpe Aliaga, James Kent, et al. 2019. "fMRIPrep: A Robust Preprocessing Pipeline for Functional MRI." *Nature Methods* 16: 111–16. https://doi.org/10.1038/s41592-018-0235-4.

Fonov, VS, AC Evans, RC McKinstry, CR Almli, and DL Collins. 2009. "Unbiased Nonlinear Average Age-Appropriate Brain Templates from Birth to Adulthood." *NeuroImage* 47, Supplement 1: S102. https://doi.org/10.1016/S1053-8119(09)70884-5.

Gorgolewski, K., C. D. Burns, C. Madison, D. Clark, Y. O. Halchenko, M. L. Waskom, and S. Ghosh. 2011. "Nipype: A Flexible, Lightweight and Extensible Neuroimaging Data Processing Framework in Python." *Frontiers in Neuroinformatics* 5: 13. https://doi.org/10.3389/fninf.2011.00013.

Gorgolewski, Krzysztof J., Oscar Esteban, Christopher J. Markiewicz, Erik Ziegler, David Gage Ellis, Michael Philipp Notter, Dorota Jarecka, et al. 2018. "Nipype." *Software*. https://doi.org/10.5281/zenodo.596855.

Greve, Douglas N, and Bruce Fischl. 2009. "Accurate and Robust Brain Image Alignment Using Boundary-Based Registration." *NeuroImage* 48 (1): 63–72. https://doi.org/10.1016/j.neuroimage.2009.06.060.

Jenkinson, Mark, Peter Bannister, Michael Brady, and Stephen Smith. 2002. "Improved Optimization for the Robust and Accurate Linear Registration and Motion Correction of Brain Images." *NeuroImage* 17 (2): 825–41. https://doi.org/10.1006/nimg.2002.1132.

Klein, Arno, Satrajit S. Ghosh, Forrest S. Bao, Joachim Giard, Yrjö Häme, Eliezer Stavsky, Noah Lee, et al. 2017. "Mindboggling Morphometry of Human Brains." *PLOS Computational Biology* 13 (2): e1005350. https://doi.org/10.1371/journal.pcbi.1005350.

Lanczos, C. 1964. "Evaluation of Noisy Data." *Journal of the Society for Industrial and Applied Mathematics Series B Numerical Analysis* 1 (1): 76–85. https://doi.org/10.1137/0701007.

Patriat, Rémi, Richard C. Reynolds, and Rasmus M. Birn. 2017. “An Improved Model of Motion-Related Signal Changes in fMRI.” *NeuroImage* 144, Part A (January): 74–82. https://doi.org/10.1016/j.neuroimage.2016.08.051.
Power, Jonathan D., Anish Mitra, Timothy O. Laumann, Abraham Z. Snyder, Bradley L. Schlaggar, and Steven E. Petersen. 2014. “Methods to Detect, Characterize, and Remove Motion Artifact in Resting State fMRI.” *NeuroImage* 84 (Supplement C): 320–41. https://doi.org/10.1016/j.neuroimage.2013.08.048.
Satterthwaite, Theodore D., Mark A. Elliott, Raphael T. Gerraty, Kosha Ruparel, James Loughead, Monica E. Calkins, Simon B. Eickhoff, et al. 2013. “An improved framework for confound regression and filtering for control of motion artifact in the preprocessing of resting-state functional connectivity data.” *NeuroImage* 64 (1): 240-56. https://doi.org/10.1016/j.neuroimage.2012.08.052.
Tustison, N. J., B. B. Avants, P. A. Cook, Y. Zheng, A. Egan, P. A. Yushkevich, and J. C. Gee. 2010. “N4ITK: Improved N3 Bias Correction.” *IEEE Transactions on Medical Imaging* 29 (6): 1310–20. https://doi.org/10.1109/TMI.2010.2046908.
Zhang, Y., M. Brady, and S. Smith. 2001. “Segmentation of Brain MR Images Through a Hidden Markov Random Field Model and the Expectation-Maximization Algorithm.” *IEEE Transactions on Medical Imaging* 20 (1): 45–57. https://doi.org/10.1109/42.906424.
